# Comparing long term impact on ovarian reserve between laparoscopic ovarian cystectomy and open laprotomy for ovarian endometrioma

**DOI:** 10.1186/1757-2215-6-76

**Published:** 2013-11-02

**Authors:** Moustafa M Zaitoun, Mohamed Moustafa Zaitoun, Manal M El Behery

**Affiliations:** 1 Obstetrics & Gynecology Departments, Faculty of Medicine, Zagazig University, Zagazig, Egypt

**Keywords:** Ovarian cysts, Laparoscopic ovarian cystectomy, Electrocoagulation, Ovarian reserve

## Abstract

**Objective:**

To compare the long term impact on ovarian reserve between laparoscopic ovarian cystectomy with bipolar electrocoagulation and laparotomic cystectomy with suturing for ovarian endometrotic cyst.

**Patient and method(s):**

121 patients with benign ovarian endometroitic cysts were randomised to either laparoscopic ovarian cystectomy using bipolar electrocoagulation (61 patients) or laparotomic ovarian cystectomy using sutures (60 patients). Serum follicle-stimulating hormone, Antimullerian hormon, Basal antral follicle Count, mean ovarian diameter, and ovarian stromal blood flow velocity were measured at 6, 12 and 18 months after surgery and compared in both groups.

**Result(s):**

A statistically significant increase of serum FSH was found in the laproscopic bipolar group at 6-, 12 and 18-month postoperativly compared to open laparotomy suture group. Also, a statistically significant decrease of the mean AMH value occurred in laproscopic bipolar group at 6-, 12 and 18-month follow- up compared to open laparotomy suture group. Basal antral follicle number, mean ovarian diameter and peak systolic velocity were significantly decreased during the 6-, 12,18 -month follow-up in laproscopic bipolar group compared to open laparotomy suture group.

**Conclusion(s):**

After laproscopic ovarian cystecomy for endometrioma all pareameter of ovarian reseve are significantly decreased on long term follow up as compared to open laprotomy.

## Introduction

There is a general consensus amongst gynecologists that ovarian endometriomas require surgical treatment due to the ineffectiveness of medical therapies [1,2]. One of the most widespread surgical techniques to excise endometriotic cysts is laparoscopic stripping. The surgical treatment of endometriomas, nevertheless, has dualistic effects on fertility: on one hand it represents a way to immediately remove the disease and reduce relapse incidence, improve symptoms like dyspareunia and improve sexual life and finally give positive effects on the chances of spontaneous conception [3]; on the other hand, it affects the so-called ovarian reserve, i.e. the pool of small antral follicles within both ovaries, potentially already compromised by the development of one or more endometriomas within the gonad [4-6].

It has been shown that removing ovarian endometriomas does not increase success rates in IVF, as it worsens the ovarian responsiveness to superovulation [5,7,8]. One key point is the surgical approach at the moment of cyst stripping: indeed, a wide variability among surgeons still exists, as part of the healthy ovarian tissue may be inadvertently excised together with the endometrioma wall [9].

Nowadays, an increasing number of young patients undergoing surgery for endometrioma are postponing fertility for many years after their treatment. It will therefore be important for these patients and their clinicians to know the possible long-term effect of endometrioma surgery on future fertility.

Short- to medium-term studies have suggested that excision of endometriomas causes significant damage to ovarian reserve and that this does not recover within up to nine months [10-14].

Despite an extensive literature search, to date we couldn’t find any study that has compared laparoscopic surgery with electro coagulation with laprotomy excision with ovarian suturing for endometrioma regarding their long term impact on ovarian reserve.

The aim of this prospective interventional study is to compare the long term impact on ovarian reserve between laparoscopic ovarian cystectomy with bipolar electrocoagulation and laparotomy cystectomy with suturing for ovarian endometrotic cyst.

## Patients and methods

This prospective randomized study was conducted from April 1, 2008, to August 31, 2012, at Zagazig University Hospitals, Zagazig, Egypt. Informed written consent was taken from each participant before enrollment in the study and the study protocol was approved by the local ethics and research committee.

The inclusion criteria were: Age 18 – 40 years; unilateral ovarian cyst with clinical and sonographic finding suggesting endometriotic cyst,; regular menstrual cycles in the previous6 months preceeding surgery.

Women who met the following criteria were excluded because these factors can affect ovarian stromal blood flow: previous ovarian surgery; surgical nessicity to perform adnexectomy, polycystic ovary syndrome according to the 2003 Rotter dam criteria [15]; or other known endocrinological disorders, history of oral contraceptive pill use or intake of other hormonal agents within 3 months before enrollment. Patients with histopathologic diagnosis of malignant ovarian cyst; or ther bengin cyst apart from endometrioma were excluded as well. Patients that got pregnant during follow period or lost follow up were also excluded.

Women with a diagnosis of unilateral ovarian cyst were observed for 3 menstrual cycles by transvaginal ultrasound examination on day 3 of each cycle to determine whether the cyst size remained the same or became bigger. Thereafter, patients were randomly allocated into two groups laparoscopy and open laparotomy groups using computer-designed randomization methods. The randomization sequence was protected (concealed) in a sealed envelope until the the operation, so that operators and patients were not aware of the assignment. The sample size was calculated to give a statistical power of 80% at a 95% confi dence interval of 1.47.

All ovarian follicles measuring 3 mm to 10 mm on both ovaries were counted preoperatively in both groups using the largest cross-sectional sagittal view of the ovary, the averaged ovarian diameters for each patient were calculated by measuring two perpendicular diameters. The stromal blood flow of the ovary was assessed by color Doppler ultrasound. Flow velocity waveforms were obtained from stromal blood vessels away from the ovarian capsule and the utero ovarian ligament. The “gate” of the Doppler was positioned when a vessel with good color signals was identified on the screen. The peak systolic velocity of stromal vessels was calculated electronically when at least three similar, consecutive waveforms of good quality were obtained.

All ultrasound studies were performed by a single experienced sonographer to decrease interob-server variability using the Voluson 370 pro V (GE Medical Systems Kretzte hnik, Zip f Austria) ultra sound device e quipped with a 7.5 MHz vaginal probe.The sample size was calculated to give a statistical power of 80% at a 95% confi dence interval of 1.47.

The serum, FSH, AMH levels were measured preoperatively on day 3 of the menstrual cycle. The DSL-10-14400 Active Müllerian-inhibiting Substance/AMH enzyme-linked immuno-sorbent assay (Diagnostic Systems Laboratories, Webster, TX, USA) was used for these measurements. The intra-assay and interassay coefficients of variation for AMH were 4.6% and 8.0%, respectively,with a detection limit of 0.017 ng/mL. All samples (preoperative andpostoperative) for a given patient were analyzed in a single assay.

In total, 79 patients underwent laparoscopic ovarian cystectomy by use of a stripping technique. After an initial laparoscopic pelvic evaluation, abdominal and peritoneal washings were performed for cytology. Laparoscopic ovarian cystectomy was performed by incision of the ovarian cyst with monopolar diathermy, identification of the cystic wall, and removal of the cyst wall from the ovarian cortex by traction with grasping forceps in opposite directions. After excision of the cyst wall, bipolar energy at a power of 40 W for 4 seconds was used to control focal bleeding. The residual ovarian tissue was not sutured, and the ovarian edges were left to heal by secondary intention.

Ovarian cystectomy by laparotomy through Pfannenstiel incision was performed on another 79 patients. After peritoneal cytology and inspection of the peritoneal cavity, the cleavage plane was developed by using microsurgical techniques and instruments. After excision of the cyst wall, meticulous reconstruction and hemostasis of the ovarian tissue were achieved by use of 2–0 polyglactin sutures (Vicryl; Ethicon Endo-Surgery, Cincinnati, OH, USA). The ovary was sutured edge-to-edge.

Frozen sections were obtained and every cyst was pathologically examined. Both techniques were performed by the same team of surgeons, with all surgeons having comparable surgical skills and experience.

All surgeries were performed within an adequate period of time. All patients were asked to return on day 3 of menstrual cycles 6, 12, 18 months after their surgery, at which point an FSH and AMH assays were performed. Basal antral follicle count, mean ovareian diameter, and peak systolic velocity of stromal vessels were also measured at 6, 12, 18 months in both groups.

Statistical analyses were performed with Statistics Package for Social Sciences software (SPSS, Inc., Chicago, IL) version 11.5 for windows. Qualitative data were expressed as number and compared using chi-squared test. Quantitative Keuls follow-up test was used for multiple comparisons between means. P < .05 was considered statistically significant.

## Results

According to the inclusion criteria, a total of 158 patients were found elligable and initially, included in the study, with 79 women being allocated to undergo laparoscopic ovarian cystectomy and 79 women being allocated to undergo open laparotomy.

Thirty seven women were excluded (4 with histopasthologic diagnosis of ovarian malignancy, 6 benign cyst other than endometrioma, 18 got pregnant during followup, 9 lost follow up). Thus, 121 women with a confirmed diagnosis of endometrioma by histopathology formed the final study group (61 patients in laproscopic group, 60 patients in laparotomy group). The general patient characteristics are presented in Table 1. Both groups were comparable in age and BMI, preoperative serun FSH, AMH were normal and comparable in both groups. Pregnancies occurred in eleven patients in open laparotomy group, and in seven patients in laparoscopic group during the 18 months follow up period which was not statically significant.

**Table 1:** Comparison between the demographic characteristics of the two studied groups

**Characteristics**	**Bipolar (N = 61)**		**Suture (N = 60)**		**T-test**	**P-value**
**Age (Years)**						
‾X±SD	24.2 ± 3.1		25.2 ± 3.0		−1.6	0.1
**BMI(kg/m**^**2)**^					14	
	27(5.8)		27(5.8)			0.2
**FSH preoperative level**	6.5 ± 0.4		6.5 ± 0.4		−0.2	0.86
**AMH preoperative level**	4.5 ± 0.8			4.6 ± 0.9	−0.1	0.8
**Pregnancy occurred**	7	4.0	11	8.0		0.7
**Preoperative AFC**	6.6 ± 2.3		6.4 ± 2.5		0.1	0.75
**Preoperative PSV**	12.7 ± 2.2	13.3 ± 1.8	1.2	0.27	1.2	0.27
**Preoperative MOD**	2.3 ± 0.6		2.4 ± 0.9		0.48	0.49

N: Number of patients.

(P > 0.05): means non-significant.

SD: Standard Deviation ‾X: Mean.

All patients had normal FSH values preoperatively. The mean values of FSH before surgery and during the 6,12,18-month follow-up period are shown in Table 2. Comparing the bipolar group with the suture group, a statistically significant increase of the mean FSH value was seen in the laproscopic bipolar group during all the 6,12,18 -month follow-up period.

**Table 2:** Comparison between the mean values of serum FSH (mIU/mL) between bipolar and suture groups

**Mean values**	**Laproscopy bipolar group (N = 61)**	**Laprotomy suture group (N = 60)**	**T-test**	**P-value**
**FSH preoperative level**				
‾X±SD	6.5 ± 0.4	6.5 ± 0.4	−0.2	0.86
**FSH 6th month**				
‾X±SD	11.4 ± 0.3	7.3 ± 0.4	31.7	0.000***
**FSH 12 th month**				
‾X±SD	10.7 ± 0.3	6.9 ± 0.4	33.5	0.000***
**FSH 18th month**				
‾X±SD	10.5 ± 0.3	6.7 ± 0.4	32.7	0.000***
***p-value***	0.000***	0.000***		

SD: Standard Deviation ‾X: Mean.

***p<0.005 highly significant.

All patients had normal AMH values preoperatively. The mean values of AMH before surgery and during the 6-,12,18 month follow-up period are shown in Table 3. Comparing the bipolar group with the suture group, a statistically significant decrease of the mean AMH value was seen in laproscopic bipolar group the during all the follow-up period.

**Table 3:** Comparison between the mean values of serum AMH (ng/mL) between bipolar and suture groups

**Mean values**	**Laproscopy bipolar group (N = 61)**	**Laprotomy suture group (N = 60)**	**T-test**	**P-value**
**AMH preoperative level**				
‾X±SD	4.5 ± 0.8	4.6 ± 0.9	−0.1	0.8
**AMH 6 th m**				
‾X±SD	2.4 ± 0.5	4.5 ± 0.9	−9.5	0.000***
**AMH 12 th**				
‾X±SD	2.7 ± 0.5	4.4 ± 0.9	−7.9	0.000***
**AMH 18th m**				
‾X±SD	2.5 ± 0.4	4.5 ± 0.9	−8.9	0.000***
***p-value***	0.000***	0.32		

(P > 0.05): Means non-significant.

***p<0.005 highly significant.

SD: Standard Deviation ‾X: Mean.

The basal antral follicle number, and mean ovarian diameter, and peak systolic velocity were comparable preoperatively in both group with no stastically significant difference (Table 4). At the 6,12,18 -month follow-up visits, the basal antral follicle number, peak systolic velocity, and mean ovarian diameter of the operated ovary in the bipolar group were statistically significantly decreased when compared with the suture group at the same time (Table 5).

**Table 4:** Comparison between the mean values of AFC, PSV (cm/s) and MOD (cm) on transvaginal ultrasound examinations of the un operated intact ovaries between bipolar and suture groups

**Mean preoperative values**	**Laproscopy bipolar group (N = 61)**	**Laprotomy suture group (N = 60)**	**T-test**	**P-value**
**AFC**	6.6 ± 2.3	6.4 ± 2.5	0.1	0.75
**PSV**	12.7 ± 2.2	13.3 ± 1.8	1.2	0.27
**MOD**	2.3 ± 0.6	2.4 ± 0.9	0.48	0.49

(P > 0.05): Means non-significant.

AFC: Antral Follicle Count.

PSV: Peak Systolic Velocity.

MOD: Mean Ovarian Diameter.

**Table 5:** Statistical comparison between post operative mean values of AFC, PSV (cm/s) and MOD (cm) on TVS examinations between bipolar and suture groups

**Mean values**	**Laproscopy bipolar group (N = 61)**	**Laprotomy suture group (N = 60)**	**T-test**	**P-value**
**6th month**				
**AFC**	3.0 ± 2.5	4.8 ± 2.1	0.0	0.05
**PSV**	8.4 ± 2.9	10.2 ± 3.4	4.1	0.05
**MOD**	2.2 ± 0.6	4.4 ± 0.2	1.5	0.03*
**12 month**				
**AFC**	3.6 ± 2.0	4.8 ± 2.3	3.5	0.05
**PSV**	8.0 ± 3.7	11.0 ± 2.9	9.2	0.004***
**MOD**	2.0 ± 0.5	2.6 ± 0.4	19.8	0.000***
**18 month**				
**AFC**	4.0 ± 2.6	5.9 ± 2.4	6.5	0.01*
**PSV**	7.8 ± 4.0	11.2 ± 3.8	9.9	0.003**
**MOD**	1.79 ± 0.3	2.4 ± 0.5	4.9	0.03*

(P > 0.05): Means non-significant.

*P<0.05: Significant.

**P<0.05: Significant

***P<0.005 highly significant.

AFC: Antral Follicle Count.

PSV: Peak Systolic Velocity.

MOD: Mean Ovarian Diameter.

## Discussion

Our study has demonstrated that bipolar coagulation of the ovarian parenchyma during laproscopic cystectomy for endometroitic cyst adversely affects ovarian reserve on long term follow up. Most studies on the topic of ovarian reserve after surgery are provided by infertility centers and are consequently limited by the selection of patients. We did not consider the woman’s postsurgical fertility a proper criterion for evaluating the ovarian reserve. Fertility is not the result of ovarian function alone and depends on multiple factors. Moreover, not all of our patients desired to get pregnant during the study period.

Comparing the laproscopic bipolar group with the suture group, a statistically significant increase of mean FSH value was seen in bipolar group at all of the follow-up visits. All patients had normal AMH values preoperatively.this runs in agreement with study of Streuli and co-workers which has established baseline similarities in circulating AMH in women with and without endometriomas [16,17]. Comparing the bipolar group with the suture group, a statistically significant decrease of the mean AMH value was seen in the laproscopic bipolar group during all the 18 month follow-up period.

When comparing the suture group with the bipolar group, a statistically significant decrease in basal antral follicle count and mean ovarian diameter were revealed in bipolar group during the 6,12,18 month follow-up evaluations; also a statistically significant decrease in peak systolic velocity was seen in bipolar group during all the follow- up visits. Our results disagree with those of Candiani et al. [18] who studied the antral follicle count, ovarian volume, stromal blood flow, and side of ovulation in 31 patients after laparoscopic cystectomy, but they failed to observe the reduction of stromal blood flow, this could be due to short term (3 months) follow up period. So they could not classify the possible mechanisms that caused gonad injury.

Several retrospective studies detected reduced responses to gonadotropin [19,20] with a marked reduction in the number of both dominant follicles and retrieved oocytes in the operated ovary after cystectomy [21,22]. While others have not found any adverse outcomes after ovarian cystectomy compared with controls [23,24] and reported that laparoscopic cystectomy of ovarian endometriomas did not affect ovarian response to gonadotropin stimulation, although the gonadotropin dose was higher in the cystectomy group.

Fedele et al. [1] reported that bipolar electrocoagulation of the ovarian parenchyma during laparoscopic removal of endometriotic ovarian cysts adversely affected ovarian function, this goes in line with our results, however in their study only FSH levels of endometrioma patients was checked which does not rule out the possible ovarian damage by endometriosis itself.

In cases where laparoscopic excision must be absolutely done (e.g. for relevant symptoms), alternative surgical techniques such as the combined cystectomy plus ablation [25] or the ultrasound-guided puncture with methotrexate [26] or alcohol injection [27,28] could be considered, especially in patients who are aged over 38 years or who have an already small ovarian reserve.

Recent study [29] showed that, even when performed by experienced laparoscopists with the highest level of cautiousness, the laparoscopic stripping of endometriotic cysts reduces the ovarian follicular reserve. The significant reduction of AMH after surgery confirms previous histological observations, suggesting that part of the healthy ovarian pericapsular tissue, containing primordial and preantral follicles, is removed or damaged despite every surgical effort to be atraumatic. This must be carefully considered when ovarian surgery is proposed to patients with one or more ovarian endometriomas, but no relevant symptoms besides infertility.

Shortcoming of this study is that it would have been more scientific to compare bipolar electro coagulation with hemostatic suturing using the laparoscopic route for both approaches. However, laparoscopic ovarian stripping using bipolar electro coagulation and open laparotomy using hemostatic suture are the 2 most commonly used techniques for managing benign ovarian cysts at the study hospital, and the surgeons participating in the study did not have adequate experience with laparoscopic suturing techniques as we mentioned previously [18].

The results of our study support the following observations. First, the laparoscopic excision of ovarian cysts is associated with a statistically significant reduction long term impact on ovarian reserve. Second, the damage cannot be ascribed merely to the amount of ovarian tissue removed during surgery; the damage to the ovarian vascular system by electrocoagulation is another factor. However, further studies in a larger number of patients are required to make certain judgments whether the injury is related to other factors and to ascertain which is the less harmful alternative therapeutic approach.

## Competing interests

The authors declare that they have no competing interests.

## Authors’ contributions

First author: study design, perform experiment. Second author: analyse data, collect material for writing, Third author: supply material for writing, shared in data analysis, wrote the manuscript. All authors read and approved the final manuscript.
